# The local skin cellular immune response determines the clinical outcome of sarcoptic mange in Iberian ibex (*Capra pyrenaica*)

**DOI:** 10.3389/fvets.2023.1183304

**Published:** 2023-06-01

**Authors:** Marta Valldeperes, José Enrique Granados, Valentín Pérez, Jorge Ramón López-Olvera, Arián Ráez-Bravo, Paulino Fandos, Jesús M. Pérez, Gregorio Mentaberre, Stefania Tampach, Ramón C. Soriguer, José Espinosa

**Affiliations:** ^1^Wildlife Ecology and Health Group (WE&H), Universitat Autònoma de Barcelona (UAB), Barcelona, Spain; ^2^Departament de Medicina i Cirurgia Animals, Servei d’Ecopatologia de Fauna Salvatge (SEFaS), Universitat Autònoma de Barcelona (UAB), Barcelona, Spain; ^3^Parque Nacional y Parque Natural de Sierra Nevada, Granada, Spain; ^4^Department of Animal Health-Instituto de Ganadería de Montaña (IGM), ULe-CSIC León, León, Spain; ^5^Faculty of Veterinary Science, University of León, León, Spain; ^6^Retired Researcher, Madrid, Spain; ^7^Department of Animal and Plant Biology, and Ecology, Jaén University, Campus Las Lagunillas, Jaén, Spain; ^8^Departament de Ciència Animal, Universitat de Lleida (UdL), Lleida, Spain; ^9^Estación Biológica de Doñana (CSIC), Sevilla, Spain

**Keywords:** *Capra pyrenaica*, clinical outcome, experimental infestation, Iberian ibex, immunohistochemistry, inflammatory cell, sarcoptic mange (*Sarcoptes scabiei*), skin immune response

## Abstract

**Introduction:**

Sarcoptic mange, caused by *Sarcoptes scabiei*, is a disease with implications for wildlife conservation and management. Its severity depends on the host’s local skin immune response, which is largely unknown in Iberian ibex (*Capra pyrenaica*), a mountain ungulate dramatically affected by mange. In this species, the clinical outcome of sarcoptic mange varies among individuals, and the local immune response could be key to controlling the infestation. This study aims to characterize the local cellular immune response and its relationship with the clinical outcome.

**Methods:**

Fourteen Iberian ibexes were experimentally infested with S. scabiei and six more served as controls. Clinical signs were monitored, and skin biopsies were collected from the withers at 26, 46, and 103 days post-infection (dpi). The presence and distribution of macrophages (including M1 and M2 phenotypes), T lymphocytes, B lymphocytes, plasma cells, and interleukine 10 were quantitatively evaluated using immunohistochemical techniques.

**Results:**

An inflammatory infiltrate that decreased significantly from 26 to 103 dpi was observed in all the infested ibexes. The predominant inflammatory cell population in the skin of the mangy ibexes was formed by macrophages (mainly the M2 phenotype) followed by T lymphocytes, with lower numbers of B lymphocytes and plasma cells. Three clinical courses were identified: total recovery, partial recovery, and terminal stage. The inflammatory infiltrates were less pronounced in the fully recovered ibexes than in those that progressed to the terminal stage throughout the study.

**Discussion:**

The results suggest an exacerbated but effective Th1-type cellular immune response controlling mange in Iberian ibex. Furthermore, the local immune response appears to determine the variability of the clinical responses to *S. scabiei* infestation in this species. This first report on the progression of local skin immune cells is relevant not only for individuals but also for population management and conservation.

## 1. Introduction

Sarcoptic mange is an emerging parasitic transmissible disease caused by the mite *Sarcoptes scabiei* and affects humans and animals worldwide (1–4). It can cause significant declines in wildlife populations; therefore, it is relevant for wildlife conservation and management (2). In Spain, sarcoptic mange has been reported in wild carnivores (5, 6), lagomorphs (7, 8), and ungulates (9–11), but the most dramatic effects of the disease have been observed in mountain ungulates, including Cantabrian chamois (*Rupicapra pyrenaica parva*) and Iberian ibex (*Capra pyrenaica*), in which outbreaks have led to demographic declines of over 95% (12–16).

Despite sarcoptic mange having been known for a long time and the number of studies on this disease conducted in humans, domestic animals, and wildlife, its clinical course and progression remain not yet fully understood. *Sarcoptes scabiei* burrows galleries in the epidermis, feeding on host cells and lymphatic fluid (17) and inducing antigenic reactions with imbalances in Th1/Th2/Th17 immune responses (18, 19). These responses drive the pathogenesis and clinical signs of sarcoptic mange, which range from mild erythema to more severe lesions, such as dermatitis, hyperkeratosis, alopecia, and systemic signs, which sometimes eventually cause death (6, 20–23). However, two research questions regarding the immune response to *S. scabiei* in different wildlife species remain not yet fully clarified: (1) what is the effect of immunological responses on mange severity, and (2) what are the primary drivers in host–parasite interactions for both positive and negative clinical outcomes of mange? (24, 25).

The pathogenesis of sarcoptic mange has been characterized in humans, in which the local skin immune response defines the severity of the parasitosis (18, 26, 27). A Th1-mediated skin immune response with higher T CD4+ lymphocyte infiltrates leads to milder ordinary scabies, while a response skewed toward Th2 with predominantly T CD8+ lymphocyte infiltrates leads to a more severe crusted scabies (18, 26, 27). Therefore, the detailed study of the inflammatory response to *S. scabiei* in skin biopsies can reveal interspecific and/or interindividual variability in the severity of clinical signs, as well as in the development of resistance and the existence of asymptomatic carriers. This has led to the study of immunological and inflammatory local skin response to sarcoptic mange both by conventional histological techniques and by immunohistochemical methods in domestic animals such as goats (*Capra hircus*), sheep (*Ovis aries*), dogs (*Canis lupus familiaris*), and pigs (*Sus scrofa domesticus*), as well as in wildlife, including chamois (*Rupicapra* spp.), red deer (*Cervus elaphus*), wolves (*Canis lupus*), red foxes (*Vulpes vulpes*), roe deer (*Capreolus capreolus*), Iberian lynxes (*Lynx pardinus*), wild boars (*Sus scrofa*), and wombats (*Vombatus ursinus*) (6, 19, 28–35). These studies have demonstrated not only interspecific but also intraspecific differences in the local skin immune response to *S. scabiei* infestation (1), which can also vary between naturally and experimentally infested animals (28). However, skin lesions and inflammatory infiltrate also depend on the stage of infestation; therefore, to precisely histologically characterize the local inflammatory response, a representative number of histological sections taken throughout the disease course must be studied. Thus, identifying inflammatory cell populations in experimentally induced scabietic lesions during the sensitization and challenge infection phases, and characterizing the timing and intensity of the immunological response during the challenge phase, could help elucidate their role and the mechanism for the immune response in wildlife (25, 29).

The features of the humoral and cellular immune responses, both local in the skin and systemic in the bloodstream, are relevant for the course of the disease. Consequently, they also influence sarcoptic mange management at both an individual and population level, as strategies and their associated costs must be weighed against the risks, hazards, and impact of the disease on the population (36). The management of sarcoptic mange in wild Caprinae, and in Iberian ibex in particular, is challenging and controversial, with different and even contradictory measures being applied and a lack of consensus not only on the management options but even on the criteria for deciding which measure to implement (16). Understanding individual immune responses that affect population dynamics is not only relevant for individual health and welfare, but also has population, ecological, and management implications.

Although Iberian ibex has repeatedly been reported to be capable of recovering and surviving sarcoptic mange (37–39), previous studies suggest that the systemic humoral immune response is not effective at preventing the development of advanced clinical stages of sarcoptic mange and eventual death. Rather, it is a non-specific indicator of the inflammatory process associated with the disease (40–42). Conversely, also in Iberian ibex, the ability to cope with sarcoptic and survive may depend on the skin local cellular immune response rather that on the both skin local and systemic humoral immune response (1, 3, 21, 43).

Therefore, the aim of this study is to immunohistochemically characterize the cellular immune skin response of Iberian ibex to experimental infestation with *S. scabiei*, describe the progression of the inflammatory infiltrate throughout the course of the disease, and investigate the potential link between this response and the clinical outcome.

## 2. Materials and methods

### 2.1. Animals

Twenty healthy free-ranging Iberian ibexes (10 females and 10 males, aged between 1 and 11 years) were captured in the Sierra Nevada Natural Space (36° 55′- 37°10′N, 2° 56′- 3° 38′W) and the Sierras de Cazorla, Segura y Las Villas Natural Park (37° 53′-37° 88′N, 2° 53′-2°88′W), in southern Spain. The ibexes were captured and immobilized with a combination of xylazine (3.0 mg/kg) and ketamine (3.0 mg/kg) (44), using a Teleinject G.U.T 50® anesthesia gas-applicator^1^. After firing a single dart from a distance of 10–20 m, the anesthesia was maintained for approximately 15 min, at which point a first clinical inspection was conducted. The ibexes were inspected for clinical signs compatible with sarcoptic mange, and the presence of antibodies against *S. scabiei* was assessed using a validated enzyme-linked immunosorbent assay (ELISA) (42). Only ibexes negative for sarcoptic mange by both physical examination and serological diagnosis were retained for the study and transported to specific experimental facilities located in the Sierra de Huétor Natural Park (37° 18′-37° 30′N, 3° 28′-3° 47′W). The ibexes were divided into groups of four to six individuals of mixed sexes and ages. Each group was kept in a separate pen measuring 30 m^2^ with access to food and water *ad libitum*. These ibex groups remained constant throughout the entire experimental period.

This study adhered to all legal requirements and guidelines related to animal welfare and experimentation in Andalusia, Spain and Europe. The handling procedures and sampling frequency were designed to minimize stress and its impact on the health of the subjects, in accordance with European (2010/63/UE) and Spanish (R.D 53/2013) standards. The study was approved by the Ethics on Animal Welfare Committee of the University of Jaén and authorized by the Dirección General de Producción Agrícola y Ganadera of the Consejería de Agricultura, Pesca y Medio Ambiente of the Junta de Andalucía (Ref: SA/SIS/MD/ps/ October 25, 2012). The Sierra Nevada Natural Park staff also approved this study.

### 2.2. Experimental infestation

After an 8-week adaption period in the facilities, 14 of the 20 ibexes were experimentally infested with *S. scabiei*. Two cm^2^ skin fragments were attached to the withers from a naturally infested free-ranging Iberian ibex, as described previously (21). Mite density was calculated in skin pieces adjacent to those used for the infestation using a stereomicroscope after overnight digestion in 5% potassium hydroxide (KOH) solution at 40°C (45). A thermal gradient was then induced by shining a light on Petri dishes with black bottoms and transparent central areas (46). The resulting estimated dose received by each ibex was 750 ± 440 mites (mean ± standard deviation). The remaining six ibexes served as non-infested controls.

### 2.3. Clinical assessment

The clinical signs and the extension of mange-compatible skin lesions were monitored for 103 days post-infection (dpi) and classified as 0 (no visible skin lesions), 1 (focal skin lesions affecting less than 50% of the body surface), or 2 (extended skin lesions affection more than 50% of the body surface), as described previously (14). None of the control ibexes developed any lesions or clinical signs compatible with mange. Conversely, both lesions and clinical signs were observed in all the infected ibexes, in which three different clinical courses were identified: four ibexes had mild skin lesions affecting less than 50% of the body surface that healed completely before the end of the 103-day experimental period (“totally recovered”); three ibexes had progressive skin lesions spreading over 70% of the body surface, but with signs of recovery, such as hair growth, reduction of skin thickening, and skin smoothing (“partially recovered”); and seven ibexes had advanced skin lesions, including alopecia, skin desquamation, thickening and crusting, and pruritus, spreading over 70% of the body surface without signs of recovery (“terminal”) (21).

### 2.4. Skin sampling

Skin biopsies were collected from the withers of the infested ibexes at 26, 46, and 103 dpi using an 8-mm diameter biopsy punch (KRUUSE® Biopsy Punch, Langeskov, Denmark), after the interscapular region was shaved. The control ibexes were sampled from the same body region using the same procedure only at 103 dpi. Each ibex was individually restrained in a handling crush, blindfolded, and locally anesthetized with a combination of 10 mg of lidocaine hydrochloride and 0.01 mg of adrenalin (ANESVET®, Ovejero Lab, León, Spain). After the skin samples were collected, the resulting skin wounds were topically treated with Bactrovet® antiseptic sprays, composed of a mixture of micronized aluminum and silver and rosehip oil.

A total of 48 skin samples were obtained (Table 1). Each biopsy was placed into 10% neutral buffered formalin for 48–72 h, then transferred to 60% ethanol and stored at 4°C until histological analysis. The biopsies were embedded in paraffin and 4-μm thick sections were either stained with hematoxylin and eosin (H&E) for skin lesion assessment (21) or used for immunohistochemical studies.

**Table 1:** Number of skin biopsies from the Iberian ibexes experimentally infested with *Sarcoptes scabiei* and controls used for immunohistochemical analyses.

Group	Day post-infestation (dpi)			Total
	26	46	103	
Terminal	7	7	7	21
Partially recovered	3	3	3	9
Totally recovered	4	4	4	12
Control			6	6
Total	14	14	20	48

### 2.5. Immunohistochemistry

Different primary antibodies raised against antigens expressed by macrophages (Iba-1), including M1 (iNOS) and M2 (CD204) subpopulations, T lymphocytes (CD3), B lymphocytes (CD20), and plasma cells (Kappa-Lambda), as well as the cytokine interleukin 10 (IL-10), were used to quantitatively assess different cell populations that play a relevant role in the local skin immune response (Table 2).

**Table 2:** Primary antibodies and protocols used to characterize the different cellular types analyzed in the skin of the Iberian ibexes experimentally infested with *S. scabiei* and controls.

Target	Specificity (clone)	Source	Epitope unmasking	Dilution
Iba-1	Macrophage (rabbit polyclonal)	Wako®	96° 20′ buffer Dako pH6	1:2,000
CD-204	MØ Scavenger receptor A (mouse monoclonal) (clone SRA-ES) M2 marker	TransGenic Inc.® (KAL-KT022)	96° 20′ buffer Dako pH6	1:400
iNOS	Inducible nitric oxide synthase (rabbit polyclonal) M1 marker	Novus®	96° 20′ buffer Dako pH6	1:150
IL-10	Interleukin-10 (mouse monoclonal)	Biorbyt® (Orb10892)	96° 20′ buffer Dako pH9	1:100
CD3	T lymphocyte (rabbit polyclonal) (clone A0452)	Dako®	96° 20′ buffer Dako pH6	1:300
CD20	B lymphocyte (rabbit polyclonal)	Thermo® (RB9013P)	No unmasking	1:150
Kappa-Lambda	Plasma cells (rabbit polyclonal)	Dako® (0191–0193)	Proteinase K 0.05%	Kappa: 1:7,000 Lambda: 1:15,000

Heat-mediated antigen retrieval was performed on 4-μm-thick sections placed onto poly-L-lysine-coated slides, using the PT Link® system (PT-Link, Agilent®, Santa Clara, CA, USA) or proteinase K, depending on the specific antibody used (Table 2). After deparaffinization, rehydration, and drying, endogenous peroxidase was blocked by immersing the sections in 3% oxygen peroxide in methanol solution for 30 min at room temperature in the dark. The sections were then incubated with the specific primary antibodies diluted in a commercial reagent (Antibody Diluent, Agilent®, Santa Clara, CA, USA) (Table 2) overnight at 4°C in a humidified chamber. After washing, immunolabeling was performed using a ready-to-use kit EnVision System® (Agilent®, Santa Clara, CA, USA) for the appropriate monoclonal or polyclonal antibodies, and slides were incubated for 40 min at room temperature. After two washes in phosphate-buffered saline (PBS), antibody localization was determined using 3,3-diaminobenzidine (Agilent®, Santa Clara, CA, USA) as a chromogenic substrate for horseradish peroxidase (HRP) of the secondary antibody. The reaction was stopped with tap water after 3–4 min or, for plasma cells, using the commercial ImmPact® Vector® kit (Red Substrate Kit) (Vector Lab, Newark, CA, USA). Finally, the slides were counterstained with Harris’s hematoxylin. Appropriate species- and isotype-matched immunoglobulins were used as controls, including sections with an isotype control for the primary antibody and those for which the primary antibody was omitted. The same examined sections were used to determine the optimal dilution and incubation pH.

### 2.6. Evaluation of the immunostained slides

A total of 356 skin sections immunolabeled for the seven cellular markers analyzed in the 48 skin samples were evaluated, corresponding to the 20 ibexes included in the study (Table 1). Ten randomly chosen fields were selected and photographed at a magnification of 400× (Nikon® Eclipse Ci microscope, coupled with a MicrosCopiaDigital MDE3-6-3 digital camera) on each slide. The immunolabeled cells were counted on digital images using the Cell Counting add-on in ImageJ (National Institutes of Health, Bethesda, MD, USA). The average value for the 10 fields counted in each slide was calculated for each immunolabeled cell subtype. Additionally, the distribution of the immunostained cells in the different skin areas was assessed.

The immunostained cell count and distribution was independently evaluated by two of the authors (J. Espinosa and V. Pérez, the latter a European College of Veterinary Pathology Diplomate), and any discordant results were reviewed using a multiheaded microscope to reach a consensus.

### 2.7. Statistical analysis

Data normality was initially assessed for each cellular type and total cellular counts using graphical methods, skewness checks, and Kolmogorov -Smirnov tests. As all the variables did not conform to normality, non-parametric statistical methods were used.

Generalized linear mixed models (GLMMs) and linear mixed models (LMMs) were fitted using the packages “nlme,”” lme4,” “mgcv,” and “MuMIn,” depending on the distribution of the variable and using the individual identity as a random term (due to non-independency of the measures) for total inflammatory cell, total macrophage, M2 macrophage, and T lymphocyte counts. The models were selected based on the Akaike information criterion (AIC) (47). The explicative variables included in the models were dpi, the experimental group (control or infested), and clinical outcome within the infested group (recovered, partially recovered, and terminal). The effects of individual variables, such as age and sex, did not affect cell number and were considered part of the individual identity in the random term. When the interaction between the dpi and outcome was significant, different GLMMs were applied to detect specific differences between groups.

B lymphocyte and plasma cell counts were too low to perform any statistical models. Therefore, Mann–Whitney *U* tests were carried out to compare these two variables between the experimental groups (control vs. infested). Differences in B lymphocyte and plasma cell counts among dpi (26, 46, and 103 dpi) within the infested clinical outcome groups (healthy, totally recovered, partially recovered, and terminal) were assessed using non-parametric Friedman’s tests for repeated measures, whereas the differences among the three different clinical outcome groups for each dpi were assessed through Kruskall–Wallis tests and pairwise comparisons using a Wilcoxon rank sum test with Bonferroni correction (48). Finally, Spearman’s rank correlation test was applied to establish possible correlations among the different cell types analyzed.

All the statistical procedures were performed with R 4.1.2 software using the functions “ggdensity,” “stats_overlay_normal_density,” and “skewness,” and the packages “nortest,” “plotrix,” “coin,” “lme4,” and “AICcmodavg” (R Development Core Team 2022). Statistical significance was set at *p* < 0.05.

## 3. Results

### 3.1. Differences between control and infested ibexes

**Figure 1:**
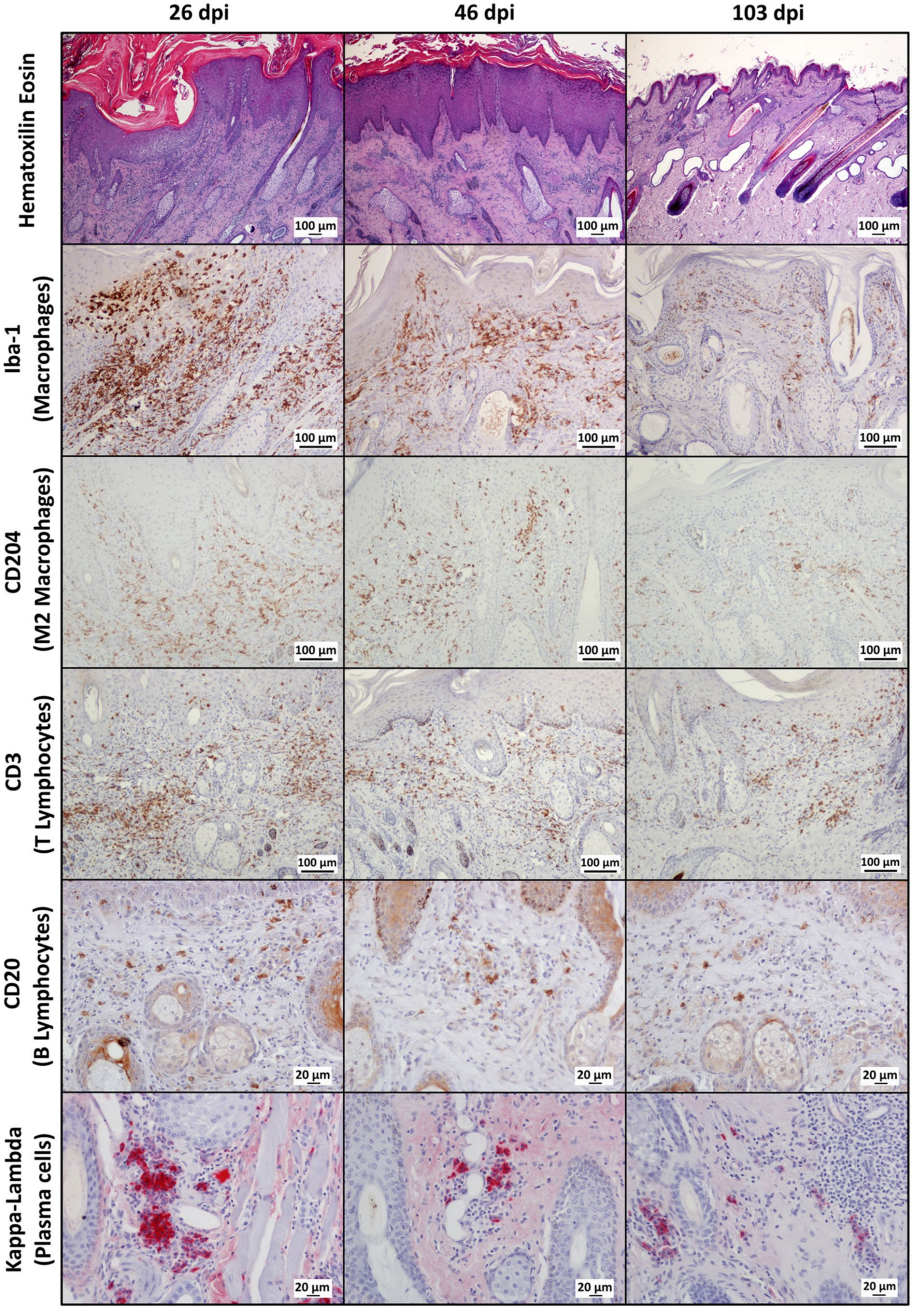
Photomicrographs of sections of mangy skin of Iberian ibexes experimentally infested with *Sarcoptes scabiei* at 26, 46, and 103 days post-infestation (dpi), showing immunolabeling of total macrophages (Iba-1 marker), the M2 macrophage subtype (CD204), T lymphocyte (CD3), B lymphocytes (CD20), and plasma cells (Kappa-Lambda). The positively immunostained macrophages (total and the M2 phenotype) and T and B lymphocytes appear brown, whereas plasma cells are red. Magnification: 200×.

Throughout the experimental infestation, five cell types (macrophages; M2 macrophages; T lymphocytes; B lymphocytes; and plasma cells) were detected in the skin of the infested (26, 46, and 103 dpi) and control ibexes (103 dpi) (Figure 1; Table 3). The identification of M1 macrophages through iNOS marker and IL-10-immunomarked cells was anecdotal. The positively immunolabeled cells were identified by their morphology and deep brown (macrophages and lymphocytes) or red (plasma cells) granular stain. When analyzing the cell counts of all the dpi altogether, all the cell types were significantly (*p* < 0.001) more abundant in the skin of the infested ibex than in the control ones. When analyzing only the samples from 103 dpi, the difference between the control and the infested ibexes was significant only for total macrophage and M2 macrophage counts (Table 3).

**Table 3:** Total cell counts for the control Iberian ibexes at 103 days post-infestation (dpi) and the infested Iberian ibexes altogether at 26, 46, and 103 dpi.

	Number of cells per field at 400× (mean ± standard error)			
	Control (103 dpi)	Infested (26 dpi)	Infested (46 dpi)	Infested (103 dpi)
Macrophages (Iba-1)	7.29 ± 1.79*	45.97 ± 3.73^a^	26.09 ± 2.02^b^	13.98 ± 2.08^c^*
M2 macrophages (CD204)	1.83 ± 0.42*	31.76 ± 2.73^a^	25.57 ± 2.17^b^	11.50 ± 2.24^c^*
T lymphocyte (CD3)	11.03 ± 0.70	33.01 ± 3.04^a^	19.11 ± 2.19^b^	15.36 ± 1.48^b^
B lymphocyte (CD20)	0.11 ± 0.06	2.66 ± 0.69^a^	1.24 ± 0.21^ab^	0.33 ± 0.09^b^
Plasma cells (Kappa-Lambda)	0.19 ± 0.10	2.84 ± 0.56^a^	2.95 ± 1.55^a^	0.26 ± 0.22^b^
Total cells	20.44 ± 2.63*	116.24 ± 8.08^a^	74.96 ± 3.64^b^	41.44 ± 4.67^c*^

a, b, and c means with different superscripts are significantly different from each other among time points within the infested Iberian ibexes; *statistically significant differences between the control and the infested Iberian ibexes at 103 dpi.

In the infested ibexes, total macrophages (Iba-1) and M2 (CD204) macrophages were observed in the superficial and intermediate dermis as well as in the epidermis, but not in the control ibexes. T lymphocytes (CD3+ immunolabeled) were observed in all the layers of the dermis, forming multifocal to confluent aggregates interspersed with macrophages. Additionally, intraepidermal foci of lymphocytic exocytosis were observed, mainly in samples from 26 dpi. Finally, antibody-producer cells (B lymphocytes and plasma cells) were less abundant in the immune cellular skin infiltrates identified in all the groups and from all the time points (Figure 1; Table 3). Both cell types were distributed mainly at the intermediate and deep dermis, but while B lymphocytes were diffusely distributed, plasma cells were seen mostly as perivascular infiltrates, mainly in the deep dermis (Figure 1).

**Figure 2:**
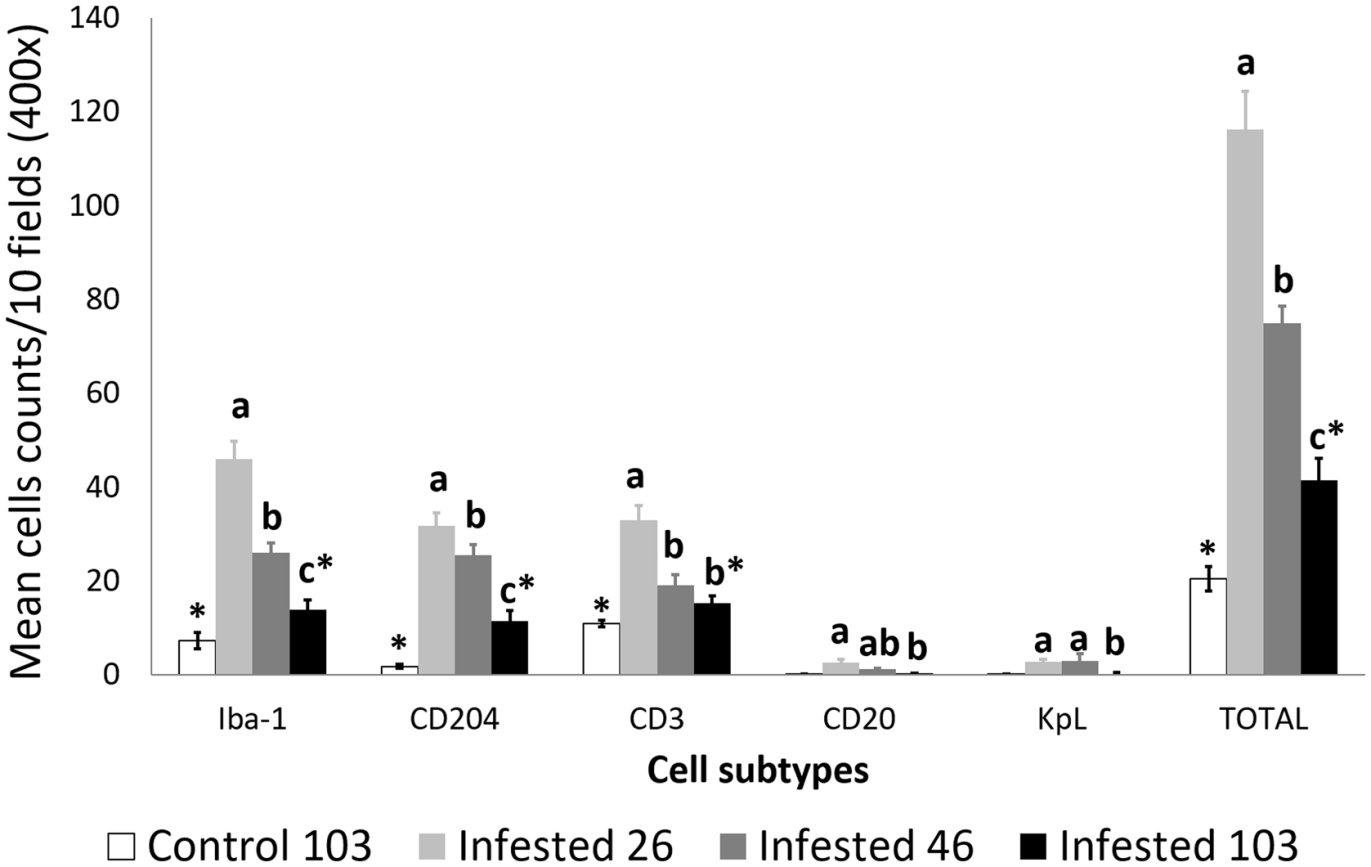
Cell counts (mean ± standard error) of positively inmunolabeled total macrophages (Iba-1 marker), M2 macrophages (CD204), T lymphocytes (CD3), B lymphocytes (CD20), and plasma cells (Kappa-Lambda) in the skin of the non-infested ibexes and the mangy skin of the infested ibexes altogether at 26, 46, and 103 days post-infestation (dpi). The total cell counts are shown as “TOTAL.” a, b, and c means with different superscripts are significantly different from each other among time points within the infested ibexes; *statistically significant differences between the control and the infested ibexes at 103 dpi.

Macrophages, both overall and especially M2, were the predominant cell type in the inflammatory infiltrate in the skin of the infested ibexes (Figure 2; Table 3). Consequently, total (Iba-1) and M2 (CD204) macrophage counts significantly correlated (*R*^2^ = 0.827, *p* < 0.001). T lymphocytes were the second most abundant cell population. Finally, B lymphocytes and plasma cell numbers were less abundant and significantly correlated (*R*^2^ = 0.707; *p* < 0.001).

### 3.2. Comparison among clinical outcomes in the infested ibexes

#### 3.2.1. Total cell counts

According to the most parsimonious model, total inflammatory cell count trend was explained by the interaction between dpi and outcome (corrected AICc [AICc] = 5072.0; degrees of freedom *K* = 10; AIC weight [AICcWt] = 1.0) (Supplementary Table S1).

The variables significantly influencing total inflammatory cell count were all the time points; the terminal outcome; the interaction of all the time points with the terminal outcome; and the interaction of 103 dpi with the partially recovered outcome. The interaction of 46 dpi with the partially recovered outcome approached significance (Table 4).

**Table 4:** Summary of the most parsimonious model explaining the evolution of total inflammatory cells in the mangy skin of Iberian ibexes by days post-infection (dpi) and outcome (recovered, partially recovered, and terminal).

Fixed effects	Estimate	SE	*z*-value	*p*-value
dpi46	−0.58	0.030	−19.45	<2.2e-16
dpi103	−1.44	0.041	−35.13	<2.2e-16
Partially recovered	−0.16	0.115	−1.36	0.175
Terminal	0.22	0.094	2.36	0.018
dpi46* partially recovered	0.09	0.047	1.85	0.064
dpi103* partially recovered	0.63	0.058	10.85	<2.2e-16
dpi46* terminal	−0.08	0.036	2.18	0.030
dpi103* terminal	0.48	0.047	10.25	<2.2e-16

SE, standard error.

Total cell counts consistently decreased throughout the study in all the infested groups (Figure 1; Supplementary Table S2). Despite this decrease, the total cell counts of the infested ibexes at 103 dpi were still higher than those of the control ibexes at 103 dpi (*p* = 1.4e-08) (Figure 2; Table 3). However, this difference from the controls was only significant for the partially recovered and terminal groups, but not for the totally recovered ibexes (Supplementary Table S3).

**Figure 3:**
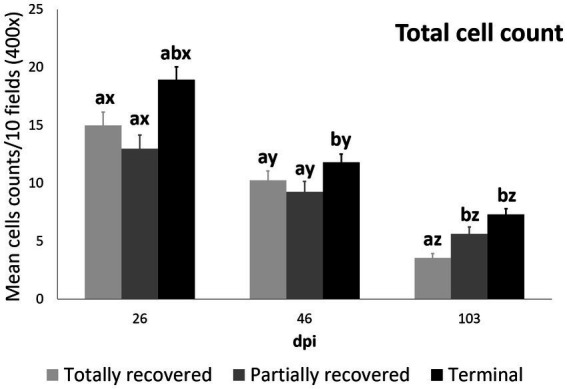
Cell counts (mean ± standard error) of the all the inmunostained cell types grouped for the clinical outcome groups at 26, 46, and 103 dpi. a and b means with different superscripts are significantly different from each other among the clinical outcome groups at the same time point; x, y, and z means with different superscripts are significantly different from each other among time points within the same clinical outcome group.

At 26 dpi, the total cell counts were higher in the terminal ibexes than in the partially recovered ones. At 46 dpi, the total cell counts of the terminal group were higher than those of the totally and partially recovered groups. At 103 dpi, the totally recovered ibexes had significantly lower total cell counts than the partially recovered and terminal groups (Figure 3; Table 3; Supplementary Table S4).

#### 3.2.2. Total macrophage counts

Total macrophage counts were also explained by the interaction between dpi and outcome, according to the most parsimonious model (AICc = 4390.2, *K* = 10, AICcWt = 1.0) (Supplementary Table S5).

The effects of all the sampling time points, all the outcomes, and the interaction of 103 dpi with all the terminal outcomes were significant on the total macrophage counts (Table 5).

**Table 5:** Summary of the most parsimonious model explaining the evolution of total macrophage counts by days post-infection (dpi) and Iberian ibex outcome (recovered, partially recovered, and terminal).

Fixed effects	Estimate	SE	*z*-value	*p*-value
dpi46	−0.53	0.046	−11.68	<2.2e-16
dpi103	−1.42	0.063	−22.45	<2.2e-16
Partially recovered	0.24	0.112	2.17	0.030
Terminal	0.58	0.092	6.34	2.3e-10
dpi46* partially recovered	−0.34	0.067	−0.58	0.565
dpi103* partially recovered	0.28	0.086	3.28	0.001
dpi46* terminal	−0.05	0.053	−0.95	0.343
dpi103* terminal	0.25	0.071	3.64	2.7e-04

SE, standard error.

**Figure 4:**
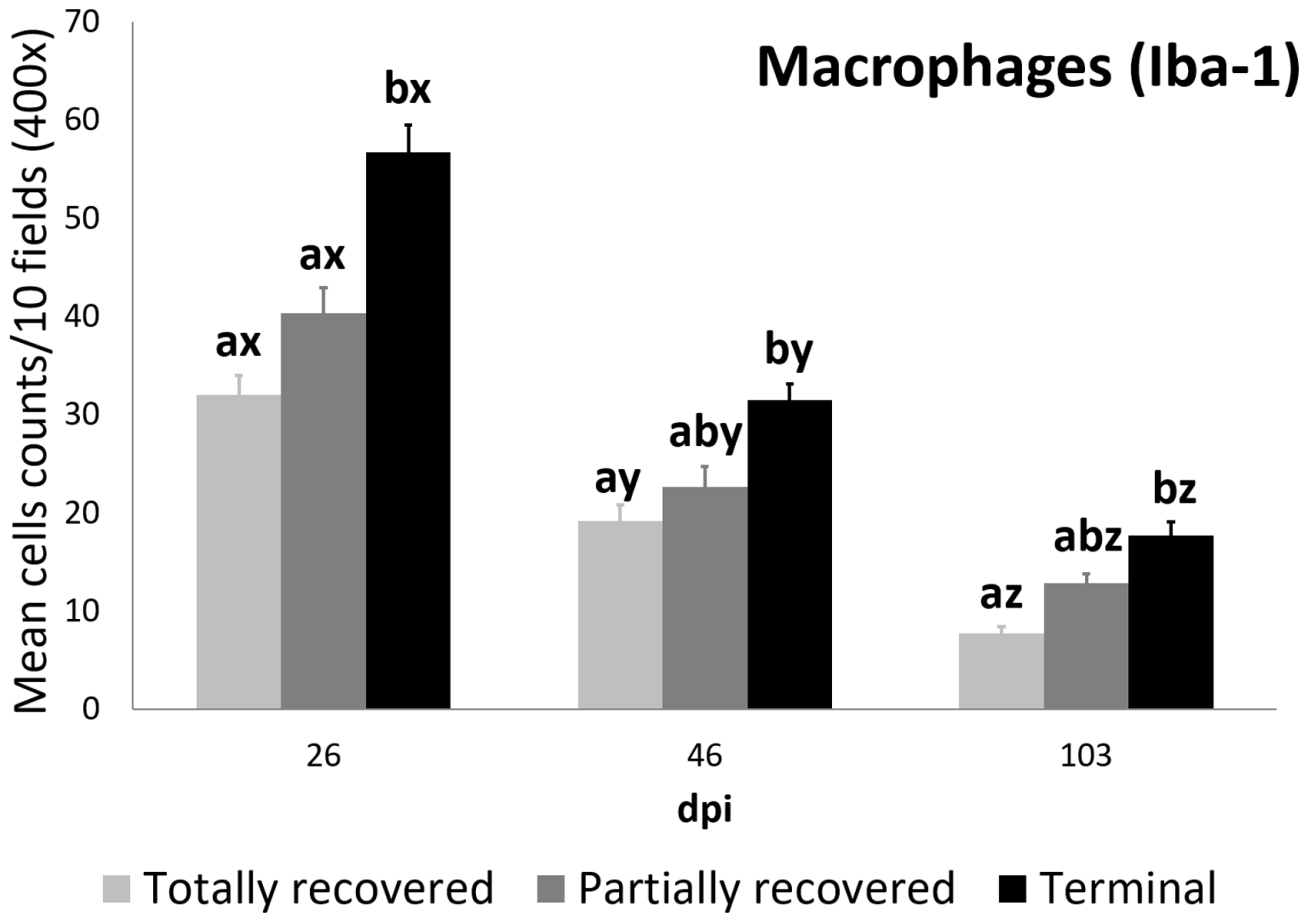
Cell counts (mean ± standard error) of positively immunolabeled Iba-1 cells (macrophages) for the clinical outcome groups at 26, 46, and 103 days post-infestation (dpi). a and b means with different superscripts are significantly different from each other among the clinical outcome groups at the same time point; x, y, and z means with different superscripts are significantly different from each other among time points within the same clinical outcome group.

In all the infected groups, the total macrophage counts consistently decreased for each time point (Supplementary Table S6; Figures 1, 4). Despite this decrease, the total macrophage counts of the infested ibexes at 103 dpi were still higher than those of the control ibexes (*p* = 5.0e-09) (Figure 2; Table 3). However, this difference from the controls was only significant for the partially recovered and terminal groups, but not for the totally recovered ibexes (Supplementary Table S7).

At 26 dpi, the total macrophage counts of the terminal ibexes were higher than both those of the totally and partially recovered groups. At 46 and 103 dpi, the total macrophage counts of the terminal ibexes were only significantly higher than those of the totally recovered group (Supplementary Table S8; Figure 4).

#### 3.2.3. M2 macrophage counts

As for total cell counts and total macrophage counts, the M2 macrophage counts were again explained by the interaction between dpi and clinical outcome, according to the most parsimonious model (AICc = 3931.3, K = 10, AICcWt = 1.0) (Supplementary Table S9).

The effects of 103 dpi, the interaction of 46 dpi with the terminal outcome, and the interactions of 103 dpi with the partially recovered and the terminal group outcomes were significant in terms of the M2 macrophage counts, while the effect of the terminal outcome approached significance (Table 6).

**Table 6:** Summary of the most parsimonious model explaining the evolution of macrophage phenotype M2 counts by days post-infection (dpi) and Iberian ibex outcome (recovered, partially recovered, and terminal).

Fixed effects	Estimate	SE	*z*-value	*p*-value
dpi46	−0.02	0.042	−0.41	0.686
dpi103	−1.42	0.068	−20.92	<2.2e-16
Partially recovered	−0.14	0.171	−0.80	0.423
Terminal	0.26	0.140	1.87	0.062
dpi46* partially recovered	0.02	0.067	0.30	0.767
dpi103* partially recovered	0.56	0.096	5.88	4.2e-09
dpi46* terminal	−0.38	0.057	−7.21	5.6e-13
dpi103* terminal	0.53	0.078	6.87	6.6e-12

SE, standard error.

**Figure 5:**
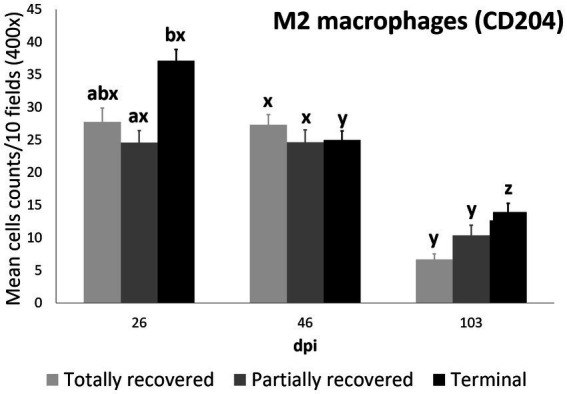
Cell counts (mean ± standard error) of positively immunolabeled CD204 cells (M2 phenotype macrophages) for the clinical outcome groups at 26, 46, and 103 days post-infestation (dpi). a and b means with different superscripts are significantly different from each other among the clinical outcome groups at the same time point; x, y, and z means with different superscripts are significantly different from each other among time points within the same clinical outcome group.

The M2 macrophage counts decreased consistently in all the study periods in the terminal group, while such a decrease was only significant for 103 dpi in the totally and partially recovered ibexes (Supplementary Table S10; Figures 1, 5). Despite these decreases, the M2 macrophage counts of the infested ibexes altogether and for each clinical outcome group were still higher than those of the control ibexes at 103 dpi (*p* = 2e-14) (Figure 2; Table 3; Supplementary Table S11).

Statistically significant differences in the M2 macrophage counts among the clinical outcomes were only found at 26 dpi, when the values of the terminal ibexes were significantly higher than those of the partially recovered group, approaching significance with the totally recovered ibexes (Supplementary Table S12; Figure 5).

#### 3.2.4. T lymphocyte counts

The T-lymphocyte count trend was also explained by the interaction between clinical outcome and dpi according to the most parsimonious model (AICc = 4587.3, K = 10, AICcWt = 1.0) (Supplementary Table S13).

The effects of all the dpi and the partially recovered outcome were significant on T lymphocyte counts, as were the interactions between the clinical outcomes and the sampling dpi (Table 7).

**Table 7:** Summary of the most parsimonious model explaining the evolution of T lymphocyte counts by days post infection (dpi) and Iberian ibex outcome (recovered, partially recovered, and terminal).

Fixed effects	Estimate	SE	*z*-value	*p*-value
dpi46	−0.87	0.046	−19.15	<2.2e-16
dpi103	−1.37	0.055	−24.92	<2.2e-16
Partially recovered	−0.66	0.171	−3.89	1.0e-04
Terminal	−0.19	0.138	−1.44	0.151
dpi46* partially recovered	0.63	0.076	8.36	<2.2e-16
dpi103* partially recovered	1.10	0.082	13.43	<2.2e-16
dpi46* terminal	0.50	0.056	8.90	<2.2e-16
dpi103* terminal	0.77	0.065	11.86	<2.2e-16

**Figure 6:**
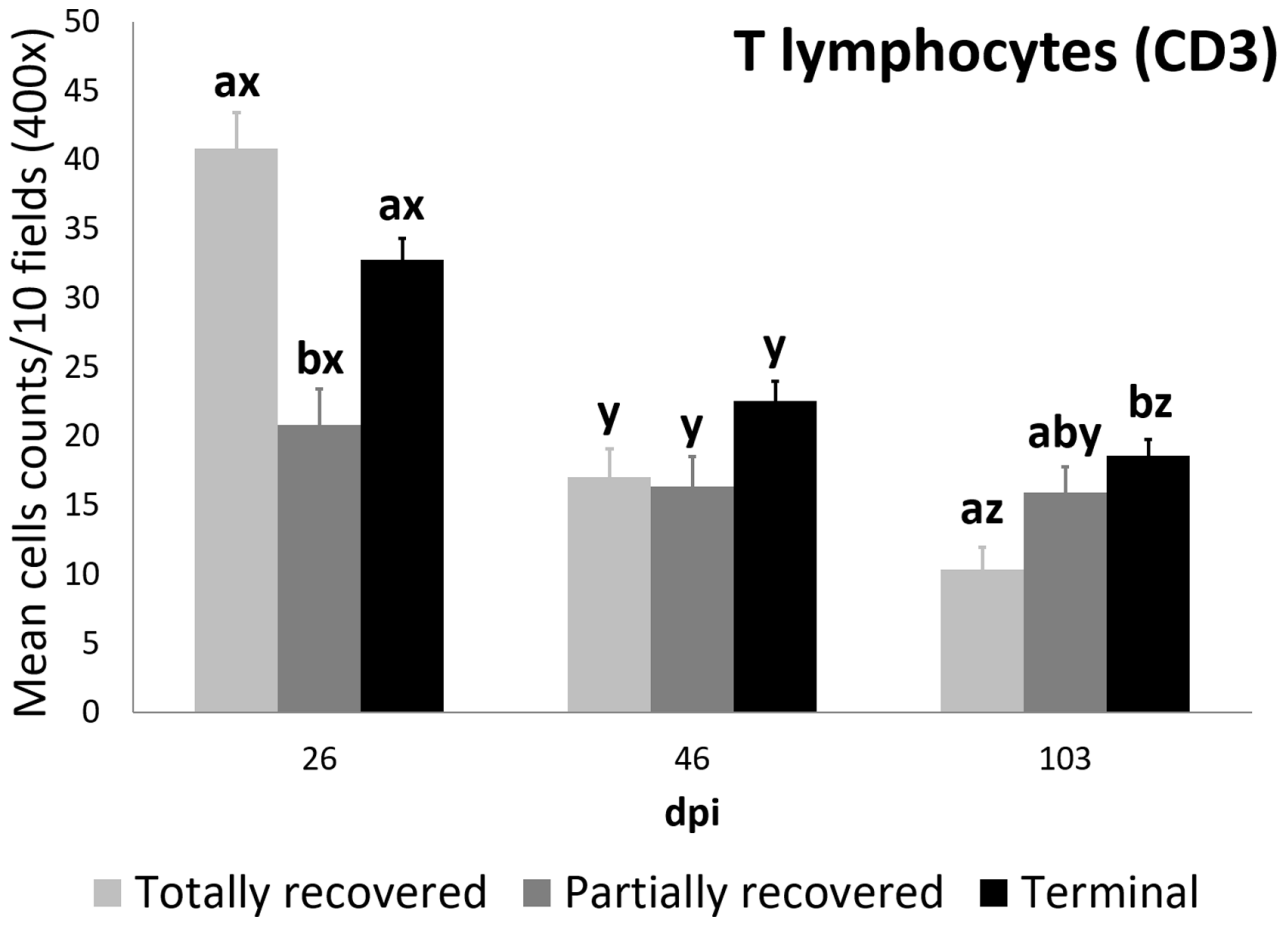
Cell counts (mean ± standard error) of positively immunolabeled CD3 cells (T lymphocytes) for the clinical outcome groups at 26, 46, and 103 days post-infestation (dpi). a and b, means with different superscripts are significantly different from each other among the clinical outcome groups at the same time point; x, y, and z, means with different superscripts are significantly different from each other among time points within the same clinical outcome group.

The T lymphocyte counts decreased consistently in each sampling interval in all the experimentally infested groups, except for the partially recovered ibexes between 46 dpi and 103 dpi (Supplementary Table S14; Figures 1, 6). The T lymphocyte counts of the infested ibexes altogether were not significantly different at any time point from those of the control ibexes at 103 dpi (Figure 2); however, the terminal group had higher T lymphocyte counts than the control ibexes at 103 dpi (Supplementary Table S15; Figure 6).

At 26 dpi, the partially recovered ibexes had lower T lymphocyte counts than the totally recovered and terminal groups. At 46 dpi, no statistically significant differences in T lymphocyte counts were detected among the three clinical outcomes. At 103 dpi, the recovered ibexes had significantly lower values than the terminal group, approaching significance versus the partially recovered ibexes (Supplementary Table S16; Figure 6).

#### 3.2.5. B lymphocyte and plasma cell counts

**Figure 7:**
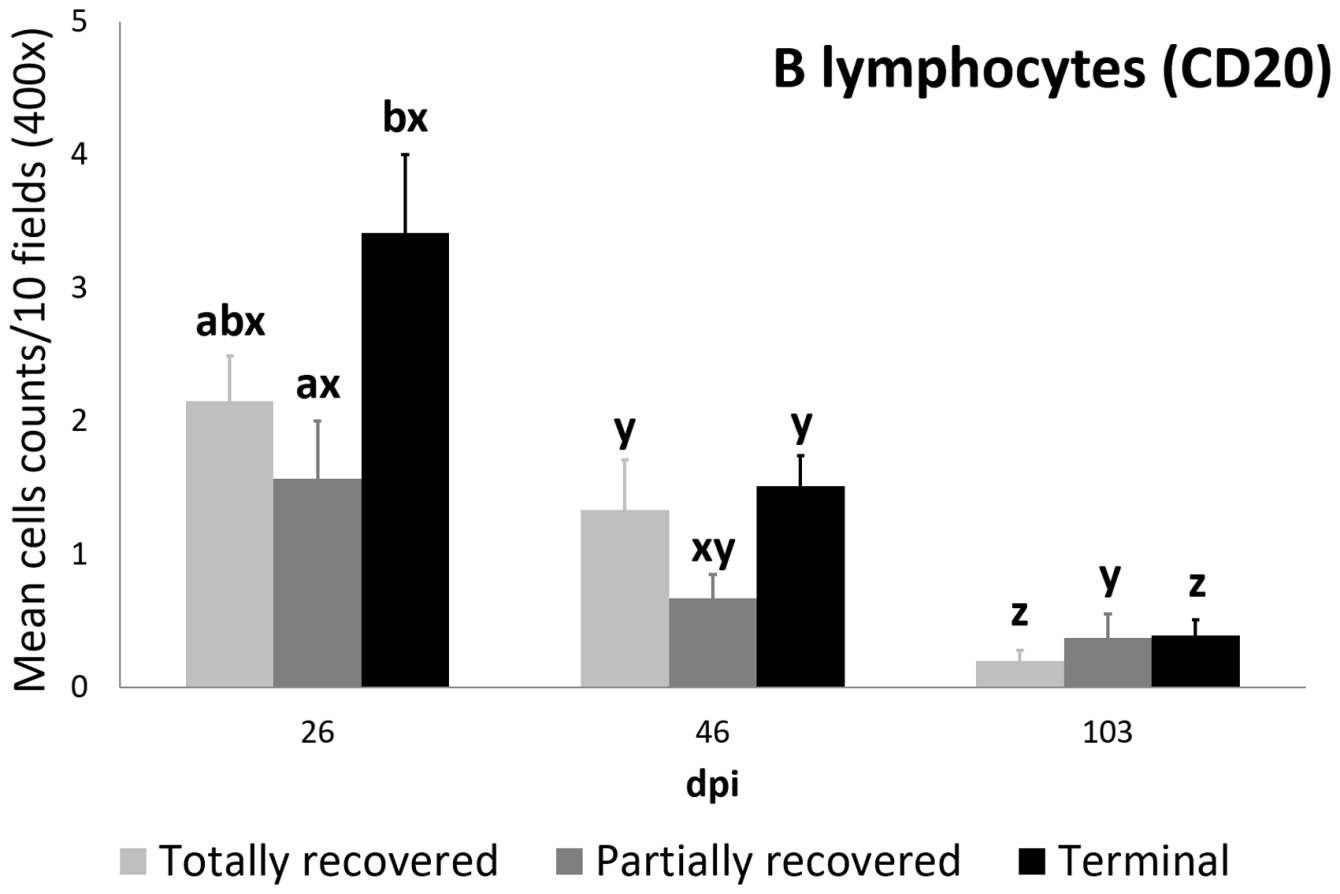
Cell counts (mean ± standard error) of positively immunolabeled CD20 cells (B lymphocytes) for the clinical outcome groups at 26, 46, and 103 days post-infestation (dpi). a and b means with different superscripts are significantly different from each other among the clinical outcome groups at the same time point; x, y, and z means with different superscripts are significantly different from each other among time points within the same clinical outcome group.

The differences in B lymphocyte counts among clinical outcomes were only significant at 26 dpi, when they were higher in the terminal than in the partially recovered ibexes (*p* = 0.023). The decrease in B lymphocyte counts was consistent in the totally recovered (26 dpi to 46 dpi *p* = 0.0499; 46 dpi to 103 dpi *p* = 0.0056) and terminal (26 dpi to 46 dpi *p* = 0.00272; 46 dpi to 103 dpi *p* = 6.2e-06) groups, while they decreased more progressively in the partially recovered ibexes (statistically significant differences were only found between 26 dpi and 103 dpi, *p* = 0.016) (Figure 7).

**Figure 8:**
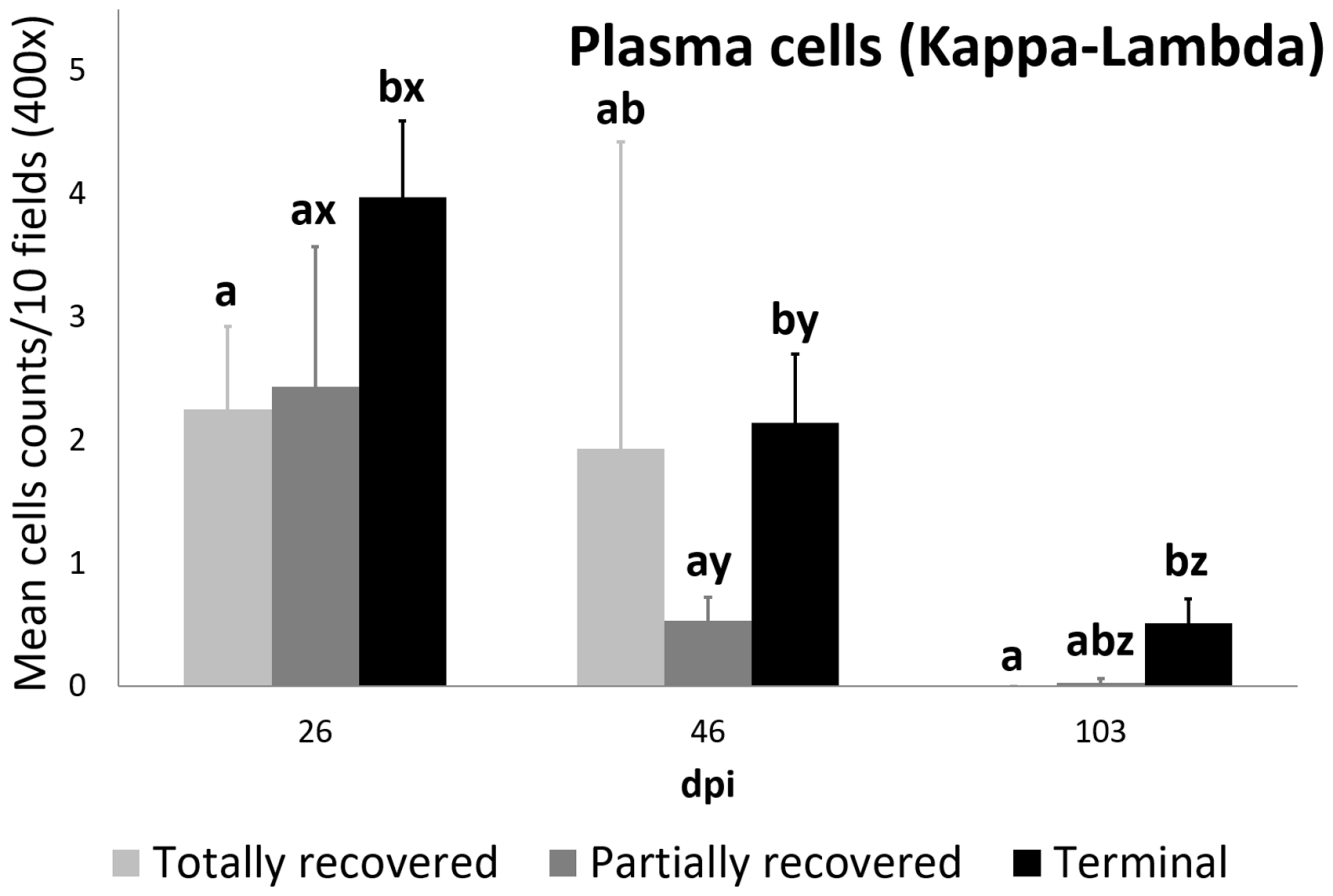
Cell counts (mean ± standard error) of positively immunolabeled Kappa-Lambda cells (plasma cells) for the clinical outcome groups at 26, 46, and 103 days post-infestation (dpi). a and b means with different superscripts are significantly different from each other among the clinical outcome groups at the same time point; x, y, and z means with different superscripts are significantly different from each other among time points within the same clinical outcome group.

Plasma cell counts were higher in the terminal group than in both the totally (*p* = 0.0450) and partially (*p* = 0.0350) recovered groups at 26 dpi. While plasma cell counts remained stable in the totally recovered group (*p* = 0.174), they decreased consistently throughout the study period in the other two groups (partially recovered: 26 dpi to 46 dpi, *p* = 0.0098; 46 dpi to 103 dpi, *p* = 0.0346; terminal: 26 dpi to 46 dpi, *p* = 0.00056; 46 dpi to 103 dpi, *p* = 0.00036). Nevertheless, the plasma cell counts of the terminal group were still higher than those of the partially recovered group at 46 dpi (*p* = 0.043) and the totally recovered group at 103 dpi (*p* = 0.026) (Figure 8).

## 4. Discussion

The capacity of Iberian ibexes to recover from and survive sarcoptic mange has been repeatedly reported (37–39). The underlying pathophysiological mechanisms of this recovery seem to rely on the local skin cellular immune response, which is driven by differences in genomic expression, rather than on any humoral immune response, either local or systemic (1, 3, 41–43, 49, 50). This longitudinal study describes for the first time the progression of the local skin immune cell response in Iberian ibexes experimentally infested with *S. scabiei*. Moreover, this is also the first report of such an analysis in any wild ungulate. More importantly, this study also provides the first evidence linking the clinical outcome of sarcoptic mange with the local skin immune cell response, specifically identifying the immune cell types involved and their relationship with the clinical outcome and eventual mortality or survival of the Iberian ibexes affected by this disease.

Compared with the control ibexes, sarcoptic mange caused a severe inflammatory response in the Iberian ibex epidermis and dermis, which can be attributed to the mechanical, excoriating, and allergenic action of the mites (51). This inflammatory infiltrate corresponded to the lesions previously reported in these same individuals, including severe hyperkeratosis, crusts, irregular epidermal hyperplasia with rete ridges, spongiotic areas with apoptotic keratinocytes, and lymphocyte exocytosis foci in the epidermis. Additionally, these lesions were related to the abundance of mites, their detritus, and galleries in the histological sections (21, 38). Such parallelism between skin lesions and inflammatory skin infiltrate has also been reported in other wildlife species, such as chamois and wombats (25, 28).

The absence of mites and the decrease of immune cells observed at 103 dpi is consistent with the previously reported tissue restoration (21) and corresponds with the decline in mite numbers (38). This reduction in mite numbers could be attributed to the exacerbated local inflammatory response observed at 26 dpi. Although this response causes severe tissue damage, it could control and eliminate the mite or promote mite displacement toward other dermal areas where the inflammatory response is still reduced (18, 19). Alternatively, the reduction of the inflammatory infiltrate throughout the dpi may not have caused the decrease in mite numbers, but rather be the consequence of mites displacing toward unaffected skin areas richer in nutritional factors (38).

This skin inflammatory response observed in the infested Iberian ibex was characterized by a predominance of macrophages (mainly the M2 subtype), followed by T lymphocytes, with a lower involvement of antibody-producing cells (B lymphocytes and plasma cells) (Figures 2–8). This type of inflammatory response is similar to that reported in other mountain ungulate and wildlife species (25, 32, 33, 52). The higher abundance of intra-epithelial macrophages in the affected ibexes compared with the control group could be due to exocytosis foci of macrophages migrating from the dermis to phagocytise the mite or increased numbers of Langerhans cells, as described in chamois and related to antigen presentation to T lymphocytes (25, 32, 33, 53).

In humans, the more severe crusted scabies is associated with fewer macrophages infiltrating the skin and an unbalanced Th1/Th17 immune response, whereas in ordinary scabies, M2 macrophages are more abundant in the inflammatory infiltrate, indicating a balanced Th1/Th2 immune response (26). Both a suppressive effect mediated by a macrophage migration inhibitory factor expressed by *S. scabiei* and/or an induction of mixed M1/M2 macrophage polarization against the scabies mite have been suggested to explain the lower macrophage counts and shift to the M2 phenotype in scabietic humans (54–56).

Although differential staining of macrophage populations (iNOS targeting M1 and CD204 targeting M2) has been attempted previously for other parasitic diseases (57–61), to the authors’ knowledge this is the first study analyzing macrophage differential expression in a parasitosis caused by an arthropod. This allowed the macrophages infiltrating the skin of the experimentally infested ibexes to be identified primarily as M2, which suppress local inflammatory response (54). Similarly, in wild carnivores, macrophages have been associated, along with neutrophils, with the milder alopecic form, interpreted as a delayed type IV hypersensitivity reaction (5, 25, 33, 62). This matches the dermis histopathology previously reported in these same Iberian ibexes, consistent with type I and type IV hypersensitivity responses (21). Overall, the consistent decrease of total and M2 macrophage counts in all the clinical outcome groups in the infested Iberian ibexes, combined with the consistently higher values in the terminal group (Figures 4, 5), indicate a successfully balanced Th1/Th2 immune response that resolves the inflammatory condition after the infestation and eliminates or displaces the mite to other areas of the body (18, 19). Such an immune response was more intense and, therefore, required a longer and more sustained response in the terminal ibexes than in either the fully or partially recovered ones (Figures 4, 5). The same explanation of a higher inflammatory response in the severely affected ibexes resolving later and more slowly than the milder increase in the totally recovered ibexes could account for the similar trend observed in T and B lymphocytes and plasma cells (Figures 6–8). To fully distinguish the local skin immune responses to sarcoptic mange determining the clinical outcome in the host, macrophage subpopulations and cytokines should be assessed and further characterized, not only in Iberian ibex but also in other species, to detect potential common inflammatory recovery patterns.

Higher counts of T lymphocytes have been described in response to sarcoptic mange in chamois, wild boars, goats, sheep, and pigs (19, 30, 32, 34, 35), whereas in foxes, wolves, red deer, and roe deer, T lymphocytes were less abundant in the inflammatory infiltrate (5, 33, 62, 63). Finally, the scarce infiltrate of B lymphocytes and plasma cells matches previous reports in mangy chamois and domestic goats and sheep (30, 34, 62). Although antibodies against *S. scabiei* have been broadly used for surveys of sarcoptic mange in wildlife, their role in providing protection against the disease is unclear. In Iberian ibex they increase with clinical severity and, generally, are an indicator of the contact and intensity of the infestation rather than protection (1, 3, 43, 63, 64), corresponding to the low involvement of antibody-producing cells in the skin found in this study.

Despite the common decreasing trend in all the cellular types observed in all three clinical outcome groups (totally recovered, partially recovered, and terminal), the differences among these three groups indicate a correlation between the local skin cellular immune response and the clinical outcome. The skin immune response to sarcoptic mange varies among species and individuals, although it usually corresponds to a type I or type IV hypersensitivity response. In the type IV hypersensitivity response, the memory of T lymphocytes seems to play a key role in limiting infection at the initial phase (18, 24, 25). Moreover, the type of T cells involved (CD4+ or CD8+) and the proportion of macrophages in the local skin immune cellular response is a differential trait between ordinary and crusted scabies in humans (26, 27). Similarly, in domestic goat and sheep, the T lymphocyte subpopulations shift to CD4+ over CD8+ lymphocytes in the local skin immune response to sarcoptic mange (30, 34). Thus, the higher initial (26 dpi) T lymphocyte count of the totally recovered ibexes (Figure 6), leading to lower total cell, macrophage (both total and the M2 phenotype), T lymphocyte, and plasma cell counts at the end of the study period (103 dpi) than in the terminal ibexes (Figures 3–6, 8), seems to confirm that an initial effective local skin T-cell immune response is crucial for controlling the spread of sarcoptic mange in Iberian ibex. Further characterization of this T-cell immune response, including the cell types, cytokines, and gene expression involved, should help us to fully understand the pathophysiological and immune mechanisms responsible for the control or extension of sarcoptic mange in Iberian ibex (21, 24–26, 49, 50, 54).

To summarize, this study reports for the first time the progression of the local skin immune cell response over time, not only to *S. scabiei* infestation in Iberian ibex but to any ectoparasite in any wild ungulate species. The skin immune cell infiltrate elicited by mange in Iberian ibex, predominantly formed by macrophages and T lymphocytes, indicates a locally intense cellular inflammatory response. The evolution over time of mite presence and the inflammatory response must be considered when assessing mange lesions through skin biopsies, as false negative results could occur due to mite absence and the complete restoration of tissue integrity as soon as 3 months after the establishment of the mite (21, 49).

The M2 macrophage phenotype predominantly induced by *S. scabiei* has immunoregulatory effects and a reparative action, which could contain the mite, limit the inflammatory infiltrate, and aid in restoring tissue integrity. However, the decrease in inflammatory infiltrate could instead be a consequence of mite displacement to unaffected skin areas, rather than a direct effect of the M2 macrophages. The higher initial T lymphocyte counts in the ibexes that had a milder clinical outcome and completely healed suggest that this early T lymphocyte immune response plays a key role in controlling the spread and severity of sarcoptic mange in this species, limiting the inflammatory infiltrate and leading to a faster resolution of the disease. Moreover, even if this first antigen-presenting response fails, differences in the local inflammatory response and consequently in the clinical outcome occur, with some individuals recovering and healing and others progressing to severe stages of the disease that ultimately lead to death.

Therefore, the development of severe sarcoptic mange and eventual death requires a sequence of different conditions. Firstly, the ibex must come into contact with an effective infective dose of *S. scabiei* mites to become infested. Secondly, the local immune skin response, which involves antigen presentation by T lymphocytes and is described in the totally recovered ibexes in this study, must fail to control the spread of the disease. Thirdly, the inflammatory response must be intense and not resolve or heal spontaneously, unlike the findings in the partially recovered ibexes in this study. Finally, the uncontrolled infestation must spread and become severe enough to exhaust the pathophysiological resources of the host.

Further investigation is necessary to gain a comprehensive understanding of the mechanisms underlying resistance to sarcoptic mange in Iberian ibex, as reported in this study. Immunological, immunohistochemical, genetic, and genomic research would be particularly informative in this regard. The present findings contribute to the growing body of evidence refuting the previously held belief that *S. scabiei* infestation in this species inevitably leads to mortality. Therefore, these findings have significant implications not only for individual disease outcomes but also for the management of the Iberian ibex populations affected by sarcoptic mange.

## Data availability statement

The original contributions presented in the study are included in the article/Supplementary material, further inquiries can be directed to the corresponding author.

## Ethics statement

The animal study was reviewed and approved by the Ethics on Animal Welfare Committee of the University of Jaén and authorized by the Dirección General de Producción Agrícola y Ganadera of the Consejería de Agricultura, Pesca y Medio Ambiente of the Junta de Andalucía (Ref: SA/SIS/MD/ps/ October 25, 2012).

## Author contributions

MV analyzed the data, drafted the original manuscript, and elaborated the final version of the manuscript. JG designed the experiment, obtained funding, performed the experimental infestation, obtained and curated the samples, and critically revised the manuscript. VP performed the immunohistopathological analyses, and critically revised the manuscript. JL-O designed the experiment, obtained funding, performed the experimental infestation, obtained and curated the samples, drafted the original manuscript, and elaborated the final version of the manuscript. AR-B performed the experimental infestation, obtained and curated the samples, and critically revised the manuscript. PF, JP, and GM designed the experiment, obtained funding, and critically revised the manuscript. ST participated in data analysis, and critically revised the manuscript. RS designed the experiment, obtained funding, and critically revised the manuscript. JE performed the experimental infestation, obtained and curated the samples, performed the immunohistopathological analyses, drafted the original manuscript, and elaborated the final version of the manuscript. All authors contributed to the article and approved the submitted version.

## Funding

This project was funded by the Consejería de Medio Ambiente de la Junta de Andalucía (project 173/2009/M/00;03/15/M/00; 861_11_M_00 and 2016/00014/M) and the Spanish Ministerio de Economía y Competitividad (projects CGL2012-40043-C02-01, CGL2012-40043-C02-02, and CGL2016-80543-P). The authors’ research activities are partially supported by the Plan Andaluz de Investigación (RNM-118 group). MV is supported by a FI-GENCAT Fellowship (2020_FI_B2_00049, which is cofinanced by the Agència de Gestió d’Ajuts Universitaris i de Recerca and the European Social Fund). GM is a Serra Húnter Fellow.

## Conflict of interest

The authors declare that the research was conducted in the absence of any commercial or financial relationships that could be construed as a potential conflict of interest.

## Publisher’s note

All claims expressed in this article are solely those of the authors and do not necessarily represent those of their affiliated organizations, or those of the publisher, the editors and the reviewers. Any product that may be evaluated in this article, or claim that may be made by its manufacturer, is not guaranteed or endorsed by the publisher.

## Footnotes

^1^ https://www.telinject.de/en/products/vario-product-line/g-u-t-50/

## Abbreviations

AIC: Akaike’s information criterion
AICc: corrected Akaike’s information criterion
AICWt: Akaike’s information criterion weight
CD4: Cluster of differentiation 4
CD8: Cluster of differentiation 8
CD3: Cluster of differentiation 3
CD20: Cluster of differentiation 20
CD204: Cluster of differentiation 204
Dpi: Days post-infection/infestation
ELISA: Enzyme linked immunosorbent assay
GLMM: General linear mixed model
H&E: Hematoxylin and Eosin
HRP: Horseradish peroxidase
Iba-1: ionized calcium-binding adapter molecule 1
IL: interleukin
iNOS: inducible nitric oxide synthase
K: degrees of freedom
KOH: potassium hydroxide
LMM: linear mixed model
PBS: phosphate buffered saline
Th1: type 1 T helper
Th2: type 2 T helper
Th17: type 17 T helper
