# Supplementary material for: The local skin cellular immune response determines the clinical outcome of sarcoptic mange in Iberian ibex (Capra pyrenaica)
Source: Front Vet Sci. 2023 Jun 1;10:1183304. doi: 10.3389/fvets.2023.1183304 (PMC10267361; doi:10.3389/fvets.2023.1183304)
Supplement: Supplementary file 1 [file Table_1.pdf]

Table S1. Summary of the AICc of the models for the total inflammatory cell counts. Outcome: recovered, partially recovered and terminal ibexes; Dpi: days post infection; K: Degrees of freedom; AICc: Corrected Akaike's Information Criterion; ΔAICc: Increase of AICc; AICcWt: AICc weight.

Model	K	AICc	ΔAICc	AICcWt
**Dpi * Outcome**	10	5072.00	0.00	1
**Dpi + Outcome**	6	5211.45	139.45	0
**Dpi**	4	5218.85	146.85	0
**Outcome**	4	9080.27	4008.28	0

Table S2. Summary of the models for the total inflammatory cells of each Iberian ibex's outcome by days post-infection (dpi). SE: standard error.

Fixed effects	Estimate	SE	z-value	p-value
**Recovered dpi26- dpi46**	-0.58	0.030	-19.45	<2.2e-16
**Recovered dpi26-dpi103**	-1.44	0.041	-35.13	<2.2e-16
**Recovered dpi46- dpi103**	-0.86	0.044	-19.51	<2.2e-16
**Partially recovered dpi26-dpi46**	-0.49	0.036	-13.60	<2.2e-16
**Partially recovered dpi26-dpi103**	-0.81	0.040	-20.16	<2.2e-16
**Partially recovered dpi46-dpi103**	-0.32	0.044	-7.24	4.3e-13
**Terminal dpi26-dpi46**	-0.51	0.020	-25.39	<2.2e-16
**Terminal dpi26-dpi103**	-0.96	0.023	-41.37	<2.2e-16
**Terminal dpi46-dpi103**	-0.46	0.025	-18.02	<2.2e-16

Table S3. . Summary of the model for the total inflammatory cell counts at 103 dpi by Iberian ibex's outcome including controls (comparative group). SE: standard error; dpi: days post-infection.

Fixed effects	Estimate	SE	z-value	p-value
**dpi103 Recovered**	0.01	0.152	0.04	0.971
**dpi103 Partially recovered**	0.51	0.164	3.11	0.002
**dpi103 Terminal**	0.73	0.129	5.65	1.6e-08

Table S4. Summary of the models for the total inflammatory cell counts of each dpi comparing among Iberian ibex's outcomes. SE: standard error; dpi: days post-infection.

Fixed effects	Estimate	SE	z-value	p-value
**dpi26 Recovered-Partially recovered**	-0.17	0.185	-0.912	0.376
**dpi26 Recovered-Terminal**	0.22	0.151	1.48	0.095
**dpi26 Partially recovered-Terminal**	0.39	0.167	2.36	0.019
**dpi46 Recovered-Partially recovered**	-0.07	0.092	-0.74	0.462
**dpi46 Recovered-Terminal**	0.29	0.075	3.93	8.7e-05
**dpi46 Partially recovered-Terminal**	0.36	0.082	4.37	1.3e-05
**dpi103 Recovered-Partially recovered**	0.50	0.187	2.70	0.007
**dpi103 Recovered-Terminal**	0.73	0.154	4.72	2.4e-06
**dpi103 Partially recovered-Terminal**	0.22	0.167	1.32	0.187

Table S5. Summary of the AICc of the models for the macrophage counts. Outcome: recovered, partially recovered and terminal ibexes; Dpi: days post infection; K: Degrees of freedom; AICc: Corrected Akaike's Information Criterion; ΔAICc: Increase of AICc; AICcWt: AICc weight.

Model	K	AICc	ΔAICc	AICcWt
**Dpi * Outcome**	10	4390.20	0.00	1
**Dpi + Outcome**	6	4401.43	11.23	0
**Dpi**	4	4418.17	27.98	0
**Outcome**	4	7027.90	2637	0

Table S6. Summary of the models for the macrophage counts of each Iberian ibex's outcome comparing among days post infection (dpi). SE: standard error.

Fixed effects	Estimate	SE	z-value	p-value
**Recovered dpi26-dpi46**	-0.54	0.046	-11.68	<2.2e-16
**Recovered dpi26-dpi103**	-1.42	0.063	-22.45	<2.2e-16
**Recovered dpi46- dpi103**	-0.89	0.068	-13.11	<2.2e-16
**Partially recovered dpi26-dpi46**	-0.58	0.048	-12.01	<2.2e-16
**Partially recovered dpi26-dpi103**	-1.14	0.058	-19.53	<2.2e-16
**Partially recovered dpi46-dpi103**	-0.57	0.064	-8.89	<2.2e-16
**Terminal dpi26-dpi46**	-0.59	0.027	-22.13	<2.2e-16
**Terminal dpi26-dpi103**	-1.17	0.033	-35.80	<2.2e-16
**Terminal dpi46-dpi103**	-0.58	0.036	-16.27	<2.2e-16

Table S7. . Summary of the model for the macrophage counts at 103 dpi by Iberian ibex's outcomes including controls (comparative group). dpi: days post-infection; SE: standard error.

Fixed effects	Estimate	SE	z-value	p-value
**dpi103 Recovered**	0.17	0.240	0.73	0.467
**dpi103 Partially recovered**	0.72	0.259	2.78	0.005
**dpi103 Terminal**	0.96	0.204	4.71	2.5e-06

Table S8. Summary of the models for the macrophage counts of each dpi comparing among Iberian ibex's outcomes. dpi: days post-infection; SE: standard error.

Fixed effects	Estimate	SE	z-value	p-value
**dpi26 Recovered-Partially recovered**	0.23	0.140	1.67	0.096
**dpi26 Recovered-Terminal**	0.57	0.115	5.01	5.4e-07
**dpi26 Partially recovered-Terminal**	0.34	0.126	2.72	0.007
**dpi46 Recovered-Partially recovered**	0.28	0.199	1.37	0.171
**dpi46 Recovered-Terminal**	0.60	0.163	3.67	2.4e-04
**dpi46 Partially recovered-Terminal**	0.33	0.178	1.84	0.066
**dpi103 Recovered-Partially recovered**	0.54	0.282	1.93	0.054
**dpi103 Recovered-Terminal**	0.79	0.232	3.39	7.0e-04
**dpi103 Partially recovered-Terminal**	0.24	0.252	0.96	0.337

Table S9. Summary of the AICc of the models for M2 phenotype macrophage counts. Outcome: recovered, partially recovered and terminal ibexes; Dpi: days post infection; K: Degrees of freedom; AICc: Corrected Akaike's Information Criterion; ΔAICc: Increase of AICc; AICcWt: AICc weight.

Model	K	AICc	ΔAICc	AICcWt
**Dpi * Outcome**	10	3931.34	0.00	1
**Dpi + Outcome**	6	4095.76	164.43	0
**Dpi**	4	4096.79	165.45	0
**Outcome**	4	5451.11	1519.77	0

Table S10. Summary of the models for the M2 phenotype macrophage counts of each Iberian ibex's outcome comparing among dpi. dpi: days post infection; SE: standard error.

Fixed effects	Estimate	SE	z-value	p-value
**Recovered dpi26-dpi46**	-0.02	0.043	-0.41	0.686
**Recovered dpi26-dpi103**	-1.42	0.068	-20.92	<2.2e-16
**Recovered dpi46- dpi103**	-1.42	0.068	-20.92	<2.2e-16
**Partially recovered dpi26-dpi46**	0.00	0.052	0.05	0.959
**Partially recovered dpi26-dpi103**	-0.86	0.068	-12.73	<2.2e-16
**Partially recovered dpi46-dpi103**	0.86	0.067	12.77	<2.2e-16
**Terminal dpi26-dpi46**	-0.40	0.031	-12.83	<2.2e-16
**Terminal dpi26-dpi103**	-0.89	0.038	-23.60	<2.2e-16
**Terminal dpi46-dpi103**	-0.49	0.040	-12.29	<2.2e-16

Table S11. Summary of the model for the M2 phenotype macrophage counts at 103 dpi by Iberian ibex's outcomes including controls (comparative group). dpi: days post infection; SE: standard error.

Fixed effects	Estimate	SE	z-value	p-value
**dpi103 Recovered**	1.37	0.436	3.16	0.002
**dpi103 Partially recovered**	1.65	0.475	3.49	4.9e-04
**dpi103 Terminal**	2.07	0.378	5.47	4.5e-08

Table S12. Summary of the models for the M2 phenotype macrophage counts of each dpi comparing between Iberian ibex's outcomes. dpi: days post infection; SE: standard error.

Fixed effects	Estimate	SE	z-value	p-value
**dpi26 Recovered-Partially recovered**	-0.12	0.188	-0.66	0.507
**dpi26 Recovered-Terminal**	0.28	0.153	1.81	0.071
**dpi26 Partially recovered-Terminal**	0.40	0.169	2.37	0.018
**dpi46 Recovered-Partially recovered**	-0.09	0.301	-0.32	0.751
**dpi46 Recovered-Terminal**	-0.14	0.247	-0.60	0.547
**dpi46 Partially recovered-Terminal**	-0.05	0.272	-0.20	0.845
**dpi103 Recovered-Partially recovered**	0.28	0.517	0.54	0.590
**dpi103 Recovered-Terminal**	0.69	0.424	1.63	0.103
**dpi103 Partially recovered-Terminal**	0.41	0.466	0.88	0.377

Table S13. Summary of the AICc of the models for T lymphocyte counts. Outcome: recovered, partially recovered and terminal ibexes groups; Dpi: days post infection; K: Degrees of freedom; AICc: Corrected Akaike's Information Criterion; ΔAICc: Increase of AICc; AICcWt: AICc weight.

Model	K	AICc	ΔAICc	AICcWt
**Dpi * Outcome**	10	4587.31	0.00	1
**Dpi + Outcome**	6	4832.84	245.54	0
**Dpi**	4	4832..96	245.65	0
**Outcome**	4	5783.08	1195.77	0

Table S14. Summary of the models for the T lymphocyte counts of each Iberian ibex's outcome comparing among days post-infection (dpi). SE: standard error.

Fixed effects	Estimate	SE	z-value	p-value
**Recovered dpi26- dpi46**	-0.87	0.046	-19.15	<2.2e-16
**Recovered dpi26-dpi103**	-1.37	0.055	-24.92	<2.2e-16
**Recovered dpi46- dpi103**	-0.50	0.062	-7.99	1.4e-15
**Partially recovered dpi26-dpi46**	-0.24	0.060	-4.00	6.3e-05
**Partially recovered dpi26-dpi103**	-0.27	0.061	-4.45	8.7e-06
**Partially recovered dpi46-dpi103**	-0.03	0.064	-0.45	0.653
**Terminal dpi26-dpi46**	-0.37	0.033	-11.43	<2.2e-16
**Terminal dpi26-dpi103**	-0.60	0.035	-17.05	<2.2e-16
**Terminal dpi46-dpi103**	-0.22	0.038	-5.92	3.2e-09

Table S15. Summary of the model for the T lymphocyte counts at 103 dpi by Iberian ibex's outcomes including controls (comparative group). dpi: days post infection; SE: standard error.

Fixed effects	Estimate	SE	z-value	p-value
**dpi103 Recovered**	-0.12	0.195	-0.60	0.550
**dpi103 Partially recovered**	0.37	0.210	1.76	0.079
**dpi103 Terminal**	0.49	0.165	2.98	0.003

Table S16. Summary of the models for the T lymphocyte counts of each dpi comparing among Iberian ibex's outcomes. dpi: days post-infection; SE: standard error.

Fixed effects	Estimate	SE	z-value	p-value
**dpi26 Recovered-Partially recovered**	-0.72	0.241	-3.00	0.003
**dpi26 Recovered-Terminal**	-0.19	0.196	-0.97	0.332
**dpi26 Partially recovered-Terminal**	0.53	0.218	2.44	0.015
**dpi46 Recovered-Partially recovered**	0.03	0.268	0.10	0.919
**dpi46 Recovered-Terminal**	0.31	0.220	1.40	0.162
**dpi46 Partially recovered-Terminal**	0.28	0.242	1.16	0.246
**dpi103 Recovered-Partially recovered**	0.49	0.267	1.84	0.066
**dpi103 Recovered-Terminal**	0.61	0.220	2.79	0.005
**dpi103 Partially recovered-Terminal**	0.12	0.239	0.512	0.609
